# Refractive Index and Alcohol-Concentration Sensor Based on Fano Phenomenon

**DOI:** 10.3390/s22218197

**Published:** 2022-10-26

**Authors:** Qiang Wang, Shubin Yan, Jilai Liu, Xiaoyu Zhang, Lifang Shen, Pengwei Liu, Yang Cui, Tingsong Li, Yifeng Ren

**Affiliations:** 1 School of Electrical and Control Engineering, North University of China, Taiyuan 030051, China; 2 School of Electrical Engineering, Zhejiang University of Water Resources and Electric Power, Hangzhou 310018, China; 3 Joint Laboratory of Intelligent Equipment and System for Water Conservancy and Hydropower Safety Monitoring of Zhejiang Province and Belarus, Hangzhou 310018, China

**Keywords:** Fano resonance, resonance splitting, sensitivity, concentration detection

## Abstract

A novel nano-refractive index sensor based on the Fano resonance phenomenon is proposed in this paper. The sensor consists of the metal-insulator-metal (MIM) waveguide and a V-ring cavity with a groove (VRCG). We analyzed the performance of the nanoscale sensor using the finite element method. The simulation results show that the asymmetry of the geometric structure itself is the main factor leading to Fano resonance splitting. In Fano splitting mode, the Fano bandwidth of the system can be significantly reduced when the sensor sensitivity is slightly reduced, so that the figure of merit (FOM) of the sensor can be substantially improved. Based on the above advantages, the sensor’s sensitivity in this paper is as high as 2765 nm/RIU, FOM = 50.28. In addition, we further applied the sensor to alcohol concentration detection. The effect is good, and the sensitivity achieves about 150. This type of sensor has a bright future in the precision measurement of solution concentrations.

## 1. Introduction

Surface plasmon polaritons (SPPs) are a type of electromagnetic wave. SPPs break the limit of diffraction size and can control light in a specific wavelength range, further promoting the micro-integration of optical devices [1,2]. The maturity of nanotechnology creates suitable conditions for the invention of nanoSPP devices, allowing SPPs to perform better in many areas: micro and nanosensors, optical switches, lithography, and so on [3,4]. In addition, there are many optical phenomena in the process of plasma coupling [5,6]. Under certain conditions, the Fano resonance phenomenon will occur, the essence of which is the interference counterbalance between the continuous bandwidth mode and the discrete narrowband mode. Because the Fano resonance waveform is very sharp and sensitive to the changes in the surrounding optical environment, it is a good option for high precision measurements [7,8,9,10].

SPPs can occur at the interface between a metal and a medium. Various structures can cause the formation of SPPs and these positively affect the MIM waveguide [11,12,13]. Based on the MIM waveguide, the system structure can be flexibly changed, and the wideband and narrowband modes of the system can be coordinated to produce the expected effect of Fano resonance [14,15,16,17]. The structure of MIM waveguide based on Fano resonance continues to receive the attention of scholars at home and abroad [18,19]. For example, Zafar et al. proposed a structure for a waveguide coupled with a pair of elliptical resonators: its final performance reached 1100 nm/RIU, and its FOM achieved more than 200 [20]. Bazgir et al. proposed a waveguide-coupled dumbbell-shaped resonator structure; their simulation sensitivity achieved 1200 nm/RIU, and its FOM reached 126 [21]. Yang et al. proposed a system consisting of a MIM waveguide with a short root and a ring cavity with a fast source, with a final sensitivity of 1420 nm/RIU and a figure of merit of 76.76 [22].

In this paper, we propose a novel coupling structure consisting of a MIM waveguide and a V-ring cavity with a groove (VRCG). Compared with the previously used single-ring design, our system sensitivity improved across an extensive range. At the same time, an asymmetric structure was added to the geometric form, which reduces the sensitivity to a small degree and effectively reduces the bandwidth of the system to significantly improve the FOM of the system. In addition, we analyzed the influence of other parameters in the design on the Fano resonance and the sensing performance of the system and, thereby, devised an ideal system structure and corresponding refractive index sensor model [23,24].

## 2. Geometric Model and Analysis Method

**Figure 1:**
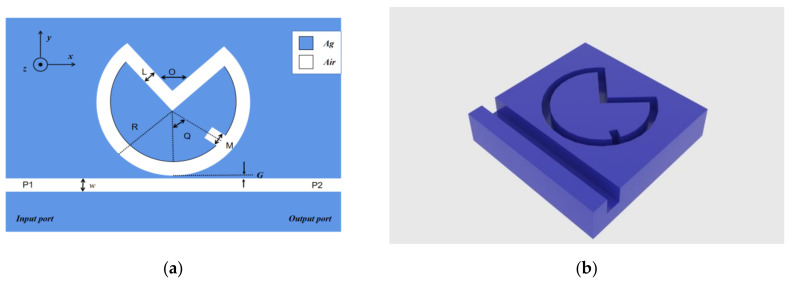
(**a**) 2D diagram of VRCG structure; (**b**) 3D schematic of the VRCG structure.

As shown in Figure 1, we modeled the system in COMSOL, with the whole system being symmetrical about the center, except for the grooves in the V-ring. Defined as shown: R is the circular outer radius, L is the distance of a V-shaped structure between up and down, O is the opening angle of V, Q is the angle of the groove center line based on the center line of the system formation, G is the spacing of the MIM waveguide coupled with the VRCG structure, M is the width of the groove, ω is the distance between the MIM waveguide; in this article, we set ω at a constant value of 50 nm.

SPPs propagate in two modes: transverse electric mode (TE) and transverse magnetic mode (TM), but only the TM mode can arouse SPPs [25]. By analyzing the TM mode, the relationship between the MIM waveguide and dispersion in this mode is shown as follows

(1) $$\tanh\left(k\omega \right)=\frac{-2kp\alpha_{c}}{k^{2}+p^{2}\alpha_{c}{}^{2}},$$

k is the wave vector, p is equal to the ratio of the dielectric constant ($\varepsilon_{in}$) and metal dielectric constant ($\varepsilon_{m}$), $\alpha_{c}={\left[k_{0}{}^{2}\left(\varepsilon_{in}-\varepsilon_{m}\right)+k\right]}^{\frac{1}{2}}$.

The analysis shows that the width of the MIM waveguide is smaller than the critical value of SPP arousal to ensure the arousal of SPPs in the TM mode [26,27,28,29]. In the 2D diagram of Figure 1a, blue and white represent silver and air, respectively, where the dielectric constant of air is set to 1. Because the dielectric constant of metal materials is affected by the incident frequency, the dielectric constant of silver was imported through the Drude–Debye mode:

(2) $$\varepsilon \left(\omega \right)=\varepsilon_{\infty }+\frac{\varepsilon_{S}-\varepsilon_{\infty }}{1+i\omega \tau }+\frac{\sigma }{i\omega \varepsilon_{0}},$$

The specific values of the parameters are as follows: relative permittivity of infinite frequency $\varepsilon_{\infty }$ = 3.8344, static permittivity $\varepsilon_{S}$ = −9530.5, relaxation time $\tau =7.35\times 10^{-15\;}s$, and conductivity $\sigma =1.1486\times 10^{7}\;s/m\;$ [30].

A suitable simulation model was selected, and the following steps were performed: (1) The excitation source is determined, and the boundary conditions are selected. (2) The VRCG structure and the waveguide structure are pre-defined as hyper-detailed in the meshing, while the remainder is pre-defined as conventional. (3) The step interval is set for the relevant calculations. (4) The simulation results are obtained for analysis. In the simulation and analysis process, we used the finite element method (FEM) to analyze and calculate the SPPs [31]. The FEM meshing is based on triangular cells [32]. Different parameters are then configured and corresponding results obtained by simulation, where sensitivity (*S*) and figure of merit (FOM) reflect the sensing performance of the structure. The specific calculation formula is as follows:

(3) $$S=\frac{\Delta \lambda }{\Delta n},$$

(4) $$FOM=\frac{S}{FWHM},$$

Above, Δλ and Δn represent the change in wavelength and refractive index, respectively, and *FWHM* is the full wave at half maximum. Both *S* and *FOM* are essential parameters for measuring sensing performance, where *S* primarily characterizes the length of the backward shift of the trough position for the exact change in refractive index, and *FOM* characterizes the extent of change in relative intensity at a given wavelength for a change in refractive index and is related to the sharpness of the Fano resonance linearity.

## 3. Simulation Results

**Figure 2:**
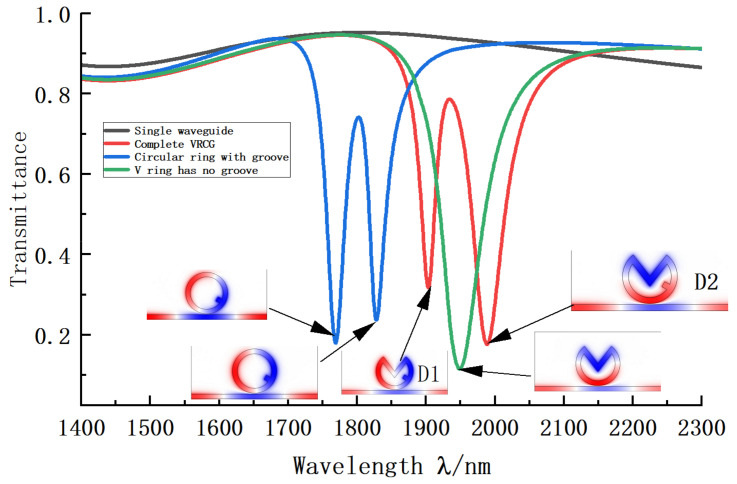
Transmission curves of complete VRCG structure (red curve), circular structure with groove (blue curve), V-ring structure without groove (green curve), single waveguide structure (black curve) Illustration: Map of the magnetic field distribution in the wave troughs indicated by the arrows.

To select a superior MIM and VRCG coupling structure, we drew the transmission curves and magnetic field distributions of the complete VRCG structure, the V-ring structure without a groove, the circular ring structure with a track, and a single waveguide. As shown in Figure 2, the radius of the ring R = 230 nm, the width of the groove M = 50 nm, the height of the groove H = 50 nm, the V-shape spacing L = 50 nm, the V-shape opening angle O = 75°, the groove angle Q = 60°, and the coupling distance between the MIM waveguide and the three structures G = 10 nm. The black MIM waveguide curve shows that the transmittance is always high and almost horizontal, so that it can be regarded as a broadband mode. However, the other three structures have different inclination positions, and are considered to be narrowband modes. Thus, it is demonstrated that all three systems arouse Fano resonance. Please note that in the subsequent analyses, the default positions of the wave valleys in the same transmission curve are D1 and D2 from left to right.

To better understand the advantages of the structure, we compared the red and blue transmission curves: it is clear that, compared with the ring structure, the V-shaped design exhibits a significant change in inclination position, and the overall curve moves to the right with an extensive range of movement, which means that the sensing sensitivity is significantly improved. By observing the magnetic field distribution, we can see that the magnetic field intensity is slightly weakened, consistent with a slight increase in transmittance. We then compared the red and green curves and found that the V-shaped ring resonator without groove demonstrates low transmission, but the system bandwidth is significant, resulting in a sharp reduction in the system figure of merit. With the addition of tracks, the Fano resonance splits due to structural asymmetry, and the shape of the coupling curve changes significantly. The final curvilinear trough case is more suitable for studying sensing performance.

**Figure 3:**
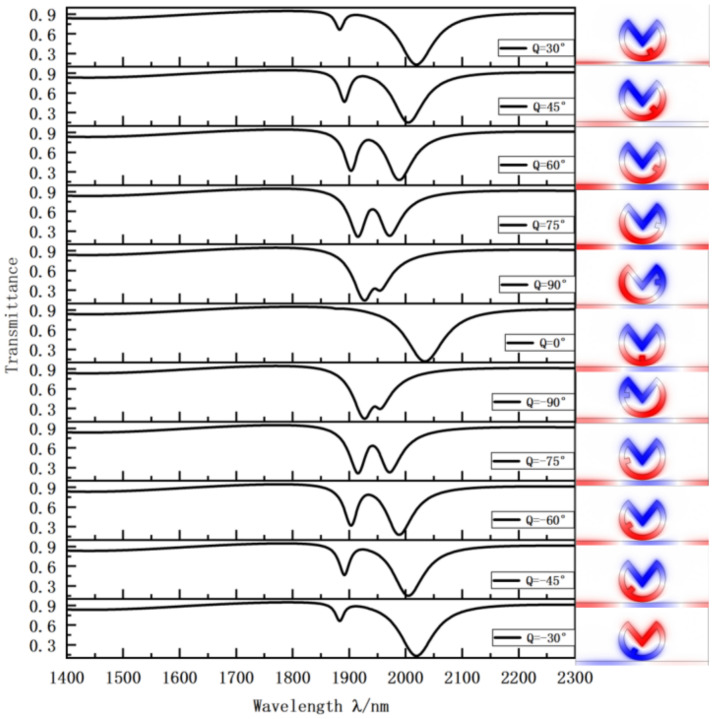
Transmission curve of VRCG structure and magnetic field distribution at D2 with varying groove angles. Inset: Plot of magnetic field intensity at the rightmost trough of the transmission curve in the same row.

Asymmetry in the VRCG structure causes Fano resonance splitting. To obtain nanostructures with a better sensing performance, we successfully drew the groove angle transmission curve changing it from negative 90 degrees to 90 degrees. The results are presented in Figure 3. The VRCG structure has different characteristics at different angles of the groove. Observing the curve when Q = 0°, we find that the transmission performance is good at this point, but the system bandwidth is large, which is not suitable for sensing performance research. From this, we altered the structure from symmetrical to asymmetrical, starting with the positive angle. From 0° to 90°, the curve continues to shift to the left. In other words, the sensitivity of sensing characteristics decreases, the transmittance increases, and the bandwidth decreases. We then observed the magnetic field intensity in the cavity under different VRCG angles and found that the structure affects the magnetic field distribution. The magnetic field intensity is more prominent in the area where the groove is located, further indicating that changing the system has little effect on the transmission but greatly improves sensitivity. In conclusion, when the groove angle is too large, the light transmission performance and sensitivity of VRCG are weakened; When the groove angle is too small, the bandwidth of the VRCG structure is too large. The curves are the same when the degrees on the left and right are the same. Thus, 0 to minus 90 degrees indicates the same characteristics. Therefore, we selected the groove angle Q = 60°, where the VRCG structure demonstrates optimal sensing performance.

**Figure 4:**
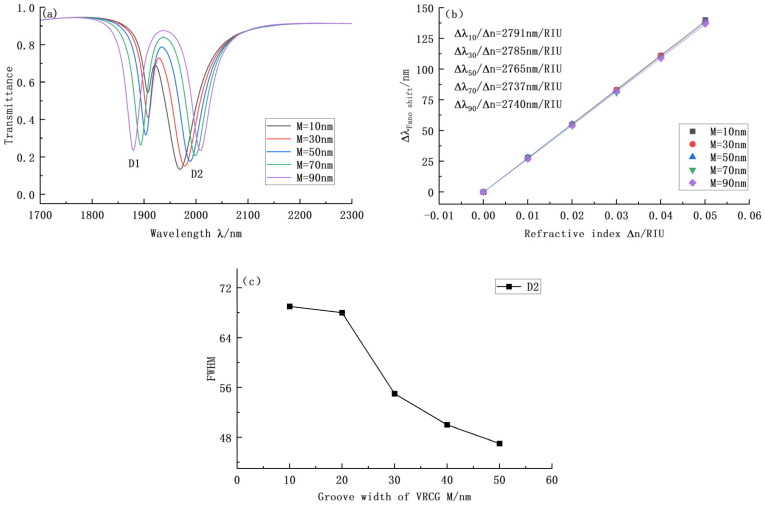
(**a**) Transmission curve of VRCG structure when the groove width changes; (**b**) Sensitivity curve fitting at D2 under different groove widths; (**c**) Variation of bandwidth at D2 under different groove widths.

In this paper, we also successfully analyze the other vital parameters affecting the VRCG structure. Figure 4a shows the transmission curve when the groove width M varies. As M increases from 10 nm to 90 nm at an interval of 20 nm, the shape of the coupling curve does not change overall, but the first trough shifts to the left, and the transmittance continues to be high; therefore, no further analysis is necessary. The position of the second dip angle continued to move to the right with the curve, but the transmission ability clearly decreased. Combined with Figure 4b, it can be seen that M has little influence on the structural sensitivity of VRCG. Finally, Figure 4c shows that the FWHM changes considerably; when combined with Figure 4c, however, when M = 70 nm and M = 90 nm, the transmittance of the system is high, so we chose M = 50 nm. Currently, the system demonstrates high sensitivity, strong transmittance, and a suitable bandwidth.

**Figure 5:**
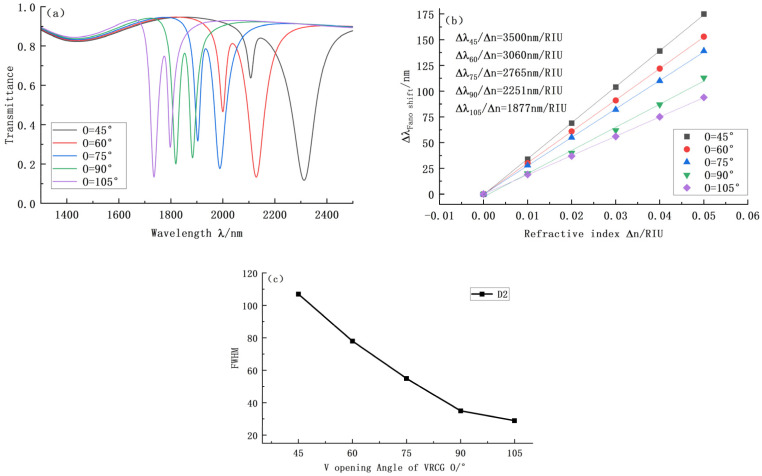
(**a**) Transmission curves of VRCG structures at different opening angles; (**b**) Sensitivity curve fitting at D2 under different opening angles; (**c**) Variation of bandwidth at D2 under different opening angles.

Figure 5 shows the corresponding transmission curve, the sensitivity at the D2 trough, and the change of its bandwidth when the angle of the opening V changes. At the D1 channel, the transmittance of 105° was ideal, so it was not compared and analyzed. Figure 5a–c shows that as the opening angle O increases, the tilt position shifts to the left, the system sensitivity decreases significantly, and the bandwidth is reduced accordingly. Finally, the FOM of different opening angles were 32.71, 39.23, 50.28, 64.31, and 64.72, respectively. Although the FOM of 105° is large, the system sensitivity is only 1877. When the opening angle is 90°, the light-limiting ability of the system deteriorates, and the transmission achieves a maximum of 0.25. When the opening angle is 45° and 60°, the corresponding FOM value of the curve is too low. To sum up, the choice of opening angle should not be too wide or too small. Finally, the opening angle O = 75° was selected in this paper.

**Figure 6:**
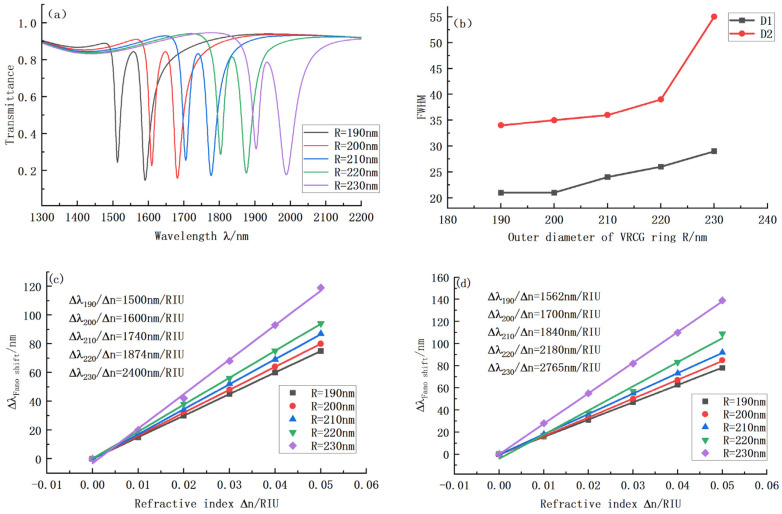
(**a**) Transmission curves for VRCG structures with different ring radii; (**b**) Variation in bandwidth at D1 and D2 under different ring radii; (**c**) Sensitivity curve fitting at D1 under different ring radii; (**d**) Sensitivity curve fitting at D2 under different ring radii.

In addition to the groove width, groove position, and the angle between the groove and the center line, the radius R of the ring is also a critical parameter affecting the structure’s performance. We successfully set the VRCG structure R as 190 nm, 200 nm, 210 nm, 220 nm, and 230 nm. The resulting images are shown in Figure 6. As is clear, the transmission curve does not change noticeably in shape with the increase in radius, but the positions of D1 and D2 uniformly move to the right, and the bandwidths at D1 and D2 increase. We successively analyzed the sensitivity and bandwidth at the two troughs and found that when R = 230 nm, the sensitivity and FOM at D1 angle position both reach the maximum in the same group, but the transmittance is higher. Therefore, D1 can be used as the sensing interval when the transmission requirement is low. D2 and FOM were 45.94, 50, 49.73, 54.56, and 50.28, respectively. The FOM values were all around 50, and the sensitivity increased successively: the sensitivity was 585 higher at R = 230 nm than at R = 220 nm. In conclusion, the radius R of the VRCG structure should be selected from an extensive range; in this paper, the V-ring radius R = 230 nm was finally selected. According to different transmission requirements, different segments can be selected for sensing applications. This is another advantage of the Fano resonance splitting mode.

**Figure 7:**
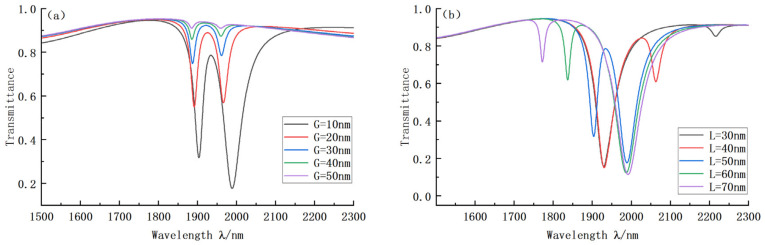
(**a**) Transmission curves of VRCG structures with different coupling spacing; (**b**) Transmission curves of VRCG structures with different V-spacing.

Finally, we studied the influence of coupling spacing G and V-shape structure distance L on the sensing performance. Figure 7a shows the transmission curves under different values of coupling distance G. It is clear that with the increase in the coupling distance, the Fano resonance phenomenon is significantly weakened, the change of G significantly changes the shape of the curve, and the light limiting the ability of the curve is seriously damaged. Therefore, it is possible to directly choose the curve with the best Fano effect, G = 10 nm. Figure 7b shows the system transmission curve when the V-shaped structure distance L changes. By analyzing the inclination position successively, it was found that when L = 50 nm, D1 can be used for sensing performance research under the condition of a low transmittance requirement. D2 can be used for sensing performance analysis under extremely high transmittance requirements. Furthermore, when L is greater than 50 nm, the corresponding D2 performance worsens. Inversely, when the distance is less than 50 nm, the D2 performance also worsens, and the performance of D1 is not as good as that of D2 when L = 50 nm. Therefore, L = 50 nm was finally selected.

**Figure 8:**
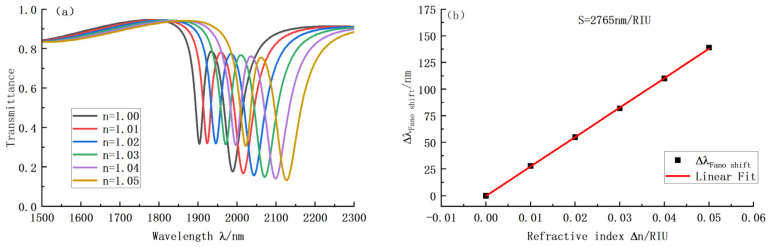
(**a**) Transmission curve of VRCG structure with varying refractive index; (**b**) Sensitivity curve fitting of VRCG structure with varying refractive index.

After all parameters were determined, we selected the optimal quantity for each parameter and finally built the complete optimized VRCG structure. The corresponding transmission and sensitivity fitting curves were drawn under different refractive indices, as shown in Figure 8. When n changes, the backward curve shifts steadily by the same distance, so the fitted curve is approximately a straight line. In our structure, the final sensitivity of the resulting VRCG structure achieves a maximum of 2765 nm/RIU, the transmission is 0.177, and the bandwidth is 55. The corresponding figure of merit is 50.28.

Finally, we plotted the sensing characteristics of this structure in comparison to the remaining designs, shown in Table 1.

## 4. Application

Currently, in certain specific situations, a partial alcohol concentration sensor is often used to monitor the production-site alcohol concentration to prevent explosion accidents. The widely used physical alcohol sensor has a high production cost and complex structure. The VRCG structure alcohol sensor described in this paper is, however, simple, cheap, and quick.

The process of VRCG monitoring of alcohol concentration is as follows: the sensor draws air at a fixed time and quantitatively. The water dissolves to produce liquid alcohol of various concentrations. When liquid enters the groove of the MIM waveguide, its refractive index is different due to the different concentrations of the intermediate medium. When the refractive index changes, the Fano resonance of the coupled structure also changes.

**Figure 9:**
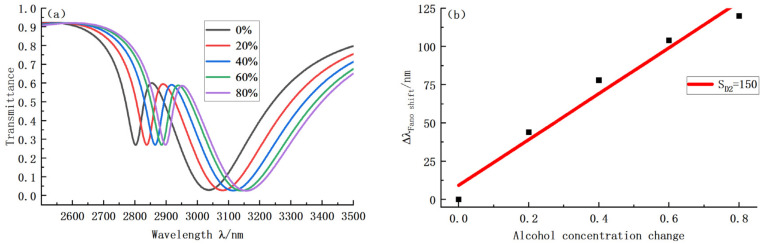
(**a**) Transmission curve with concentration change; (**b**) Sensitivity of concentration change fitting sensor.

After numerous experiments, the relationship between alcohol concentration and refractive index was obtained as follows:

At concentrations less than 50 percent,

(5) C=2054.4n−2745.1,

At concentrations less than 80 percent,

(6) C=2771.7n−3711.4,

where C is the concentration of the solution and n is the refractive index of the liquid. Based on this, we successively calculated the refractive index of the liquid when the solution concentration was 0, 20, 40, 60, and 80, respectively: 1.336, 1.346, 1.356, 1.361and 1.368. The transmission curve and sensitivity curves were finally obtained, as shown in Figure 9. Figure 9a shows that the curve has a sizeable backward shift in the early stage and a slight backward shift in the late stage, which is consistent with the sensitivity fitting result. At the same time, since the refractive index of 0 concentration solution is also as high as 1.333, the D1 trough transmission of this type of sensor is too large, so we only analyzed the sensitivity of D2. According to Figure 9b, the sensitivity of D2 reaches 150. In the later stage, the main work of this sensor is to improve measurement range and accuracy. Further measurements confirmed that the alcohol sensor has an accuracy of 0.6%, i.e., the sensor works properly at a minimum 0.6% change in concentration. In addition, we measured an accuracy of 1.25%.

## 5. Conclusions

In this study, we propose a nano-refractive index sensor structure consisting of a MIM waveguide and a V-shaped ring cavity with grooves. We obtained a perfect Fano resonance transmission curve. Previously, the traditional MIM waveguide structure was used as the broadband mode, but, here, the new VRCG structure is used as the narrow band mode. Meanwhile, the asymmetry of the VRCG structure leads to the Fano resonance splitting of the system. In split mode, the system can reduce the transmission performance in a small range, significantly reduce the system bandwidth, and finally stabilize the FOM of the system. The final sensitivity of the system reached 2765 nm/RIU, and the FOM value reached 50.28. In addition, we explored the influence of VRCG structural parameters on the sensing performance of the system, obtained the sensor with the optimal parameters, and applied it to alcohol concentration detection. Finally, the sensitivity of this type of alcohol concentration sensor was about 150. It provides a new choice for high-precision nanometer-level concentration sensors.

## Figures and Tables

**Table 1:** Comparison table of sensing characteristics of different structures.

Reference	Sensitivity	FOM	Operating Wavelength Range
[20]	1100 nm/RIU	224	1300 nm < *λ* < 1900 nm
[21]	1200 nm/RIU	126	800 nm < *λ* < 1600 nm
[22]	1420 nm/RIU	76.76	1000 nm < *λ* < 1600 nm
VRCG	2765 nm/RIU	50.28	1500 nm < *λ* < 2300 nm

## Data Availability

The data presented in this study are available on request from the corresponding author.
